# Diethylstilbestrol administration inhibits theca cell androgen and granulosa cell estrogen production in immature rat ovary

**DOI:** 10.1038/s41598-017-08780-7

**Published:** 2017-08-21

**Authors:** Yoshitaka Imamichi, Toshio Sekiguchi, Takeshi Kitano, Takashi Kajitani, Reiko Okada, Yoshihiko Inaoka, Kaoru Miyamoto, Junsuke Uwada, Satoru Takahashi, Takahiro Nemoto, Asuka Mano, Md Rafiqul Islam Khan, Md Tariqul Islam, Koh-ichi Yuhki, Hitoshi Kashiwagi, Fumitaka Ushikubi, Nobuo Suzuki, Takanobu Taniguchi, Takashi Yazawa

**Affiliations:** 1 Department of Pharmacology, Asahikawa Medical University, Hokkaido, 078-8510 Japan; 2 The Noto Marine Laboratory, Division of Marine Environmental Studies, Institute of Nature and Environmental Technology, Kanazawa University, Ishikawa, 927-0553 Japan; 3 Department of Materials and Life Science, Graduate School of Science and Technology, Kumamoto University, Kumamoto, 860-8555 Japan; 4 Department of Biochemistry, Faculty of Medical Sciences, University of Fukui, Fukui, 910-1193 Japan; 5 Department of Biological Science, Faculty of Science, Shizuoka University, Shizuoka, 422-8529 Japan; 6 Department of Biochemistry, Asahikawa Medical University, Hokkaido, 078-8510 Japan; 7 Department of Pediatrics, Asahikawa Medical University, Hokkaido, 078-8510 Japan; 8 Department of Physiology, Nippon Medical School, Tokyo, 113-8602 Japan; 9 Department of Pharmacy, University of Rajshahi, Rajshahi, Bangladesh

## Abstract

Diethylstilbestrol (DES), a strong estrogenic compound, is well-known to affect the reproductive system. In this study, we investigated the effects of DES administration on gonadotropin levels and ovarian steroidogenesis in prepubertal rats. DES treatment acutely reduced serum LH levels, followed by a reduction in the expression of various steroidogenesis-related genes in theca cells. Serum FSH levels were almost unaffected by DES-treatment, even though Cyp19a1 expression was markedly reduced. Serum progesterone, testosterone and estradiol levels were also declined at this time. LH levels recovered from 12 h after DES-treatment and gradually increased until 96 h with a reduction of ERα expression observed in the pituitary. Steroidogenesis-related genes were also up-regulated during this time, except for Cyp17a1 and Cyp19a1. Consistent with observed gene expression pattern, serum testosterone and estradiol concentrations were maintained at lower levels, even though progesterone levels recovered. DES-treatment induced the inducible nitric oxide synthase (iNOS) in granulosa cells, and a nitric oxide generator markedly repressed Cyp19a1 expression in cultured granulosa cells. These results indicate that DES inhibits thecal androgen production via suppression of pituitary LH secretion and ovarian Cyp17a1 expression. In addition, DES represses Cyp19a1 expression by inducing iNOS gene expression for continuous inhibition of estrogen production in granulosa cells.

## Introduction

In female, estrogen is mainly produced by the cooperative actions of theca and granulosa cells within the ovary. Theca cells produce androgens from cholesterol through a series of reactions catalyzed by steroid cytochrome P450 (CYP) hydroxylases and hydroxysteroid dehydrogenases under luteinizing hormone (LH) stimulation^1, 2^. The source of cholesterol for steroidogenesis primarily depends on cholesterol ester uptake from plasma proteins by lipoprotein receptors, such as scavenger receptor class B member 1 (SR-BI), although *de novo* synthesis and intracellular store also contribute to this process. Cholesterol transport from the outer to inner mitochondria membrane by steroidogenic acute regulatory protein (StAR) represents a rate-limiting step of steroidogenesis. Subsequently, steroidogenesis begins with conversion of cholesterol into pregnenolone by the cytochrome P450 side chain cleavage enzyme (CYP11A1). Pregnenolone is subsequently transferred to the cytoplasm where it is converted to androgens (androstenedione and dehydroepiandrosterone) by the catalytic actions of 3β-hydroxysteroid dehydrogenase (3β-HSD) and CYP17A1. With aid of 17β-hydroxysteroid dehydrogenases (HSD17B), these androgens are converted to estrogens by aromatase (CYP19A1) in granulosa cells. Follicle stimulating hormone (FSH) is the primary regulator of CYP19A1 and HSD17B1 expression in granulosa cells^3–5^. Upon binding to cognate receptors (LHR and FSHR, respectively), LH and FSH increase androgen and estrogen production by up-regulating gene expression of previously described steroidogenesis-related factors. This occurs mainly via transcriptional factors, such as steroidogenic factor-1 (SF-1, also known as adrenal 4 binding protein) and liver receptor homolog-1 (LRH-1)^6^.

Estrogens produced within the ovary are essential for female reproduction though regulation of the estrous or menstrual cycle. Such functions of estrogens are evoked by two different types of estrogen receptors (ERs), namely ERα and ERβ^7–9^. Female *ERα* knockout (αERKO) mice are infertile, as they exhibit anovulatory phenotype resulting from excessive LH secretion from the pituitary^10, 11^. They also display hemorrhagic cystic ovaries and ovarian steroidogenic abnormalities. Female *ERβ* knockout (βERKO) mice also exhibit a sub-fertile phenotype resulting from a lower efficiency of ovulation, caused by incomplete differentiation of granulosa cells^12, 13^. In addition to reproductive axis, estrogen/ER pathways are also involved in the development and physiology of female organs, such as mammary glands, uterus and vagina^14, 15^. As such, these organs are potential targets of endocrine disrupting chemicals (EDCs) that mimic endogenous estrogen signals.

Diethylstilbestrol (DES), a synthetic nonsteroidal compound possessing strong estrogenic activity, represents one of the best-studied EDCs across various animal species. It is evident from administration to pregnant women and animal experiments that DES has various detrimental effects to offspring during the perinatal period^16^. Prenatal DES exposure causes reproductive organ malformation and dysfunction, low fertility and reproductive tract tumors during later life^17, 18^. In animal models, these adverse effects appeared even with very low dosage of DES. On the other hand, it is well-known that large amounts of DES strongly promote the proliferation of ovarian granulosa cells and formation of preantral/early antral follicles in prepubertal rodents^19^. Indeed, various physiological phenomena of granulosa cells have been revealed from the studies using cells obtained from the ovaries of DES-primed animals^20–22^. However, the effects of DES as a potential EDC for the hypothalamic-pituitary-ovary axis are not well evaluated in this model. In this study, we investigated the effects of DES-treatment on gonadotropin secretion and ovarian steroidogenesis in immature rats. We also investigated mechanisms underlying the regulation of CYP19A1 expression by DES.

## Results

### DES affects folliculogenesis, serum gonadotropin and ovarian cognate receptor expression in immature rats

**Figure 1:**
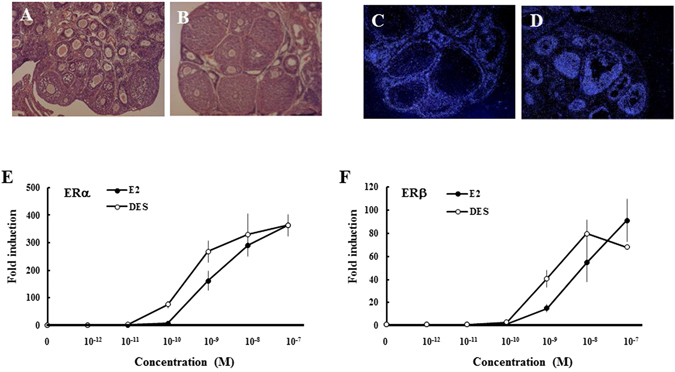
Effects of diethylstilbestrol (DES) and expression of estrogen receptors (ERs) in immature rat ovaries. Histology of immature ovaries before (**A**) or after quadruplicate DES treatment (**B**). Analyses of ERα (**C**) and ERβ (**D**) mRNA localization in rat ovaries by *in situ* hybridization. Dose-dependent effects of estradiol (E2) and DES on the transactivation of rat ERs (**E**,**F**). COS-7 cells were transfected with a 5xGAL4-E1B/Luc expression vector and an ER-encoding vector. At 24 h post-transfection, cells were incubated with or without increasing concentrations of each estrogen for 24 h. Data represent the mean ± SEM of at least four independent experiments.

Immature rat ovaries were primarily occupied by follicles at early stages (Fig. 1A). Ovarian ERα was expressed in theca and interstitial spaces, whereas ERβ expression was detectable exclusively in granulosa cells (Fig. 1C and D). Expression of ERα and ERβ mRNAs was maintained at initial levels during DES treatment, even though it was elevated at some time points (Supplementary Fig. S1). Reporter assays indicated that DES can activate both ER isoforms, in a similar manner to 17β-estradiol (E2) (Fig. 1E and F). After DES-treatment, folliculogenesis proceeded to preantral and early antral stage with multiple granulosa cell layers in many follicles (Fig. 1B), whereas it was remained at an early follicle stage in sesame oil-injected animals (Supplementary Fig. S2).

**Figure 2:**
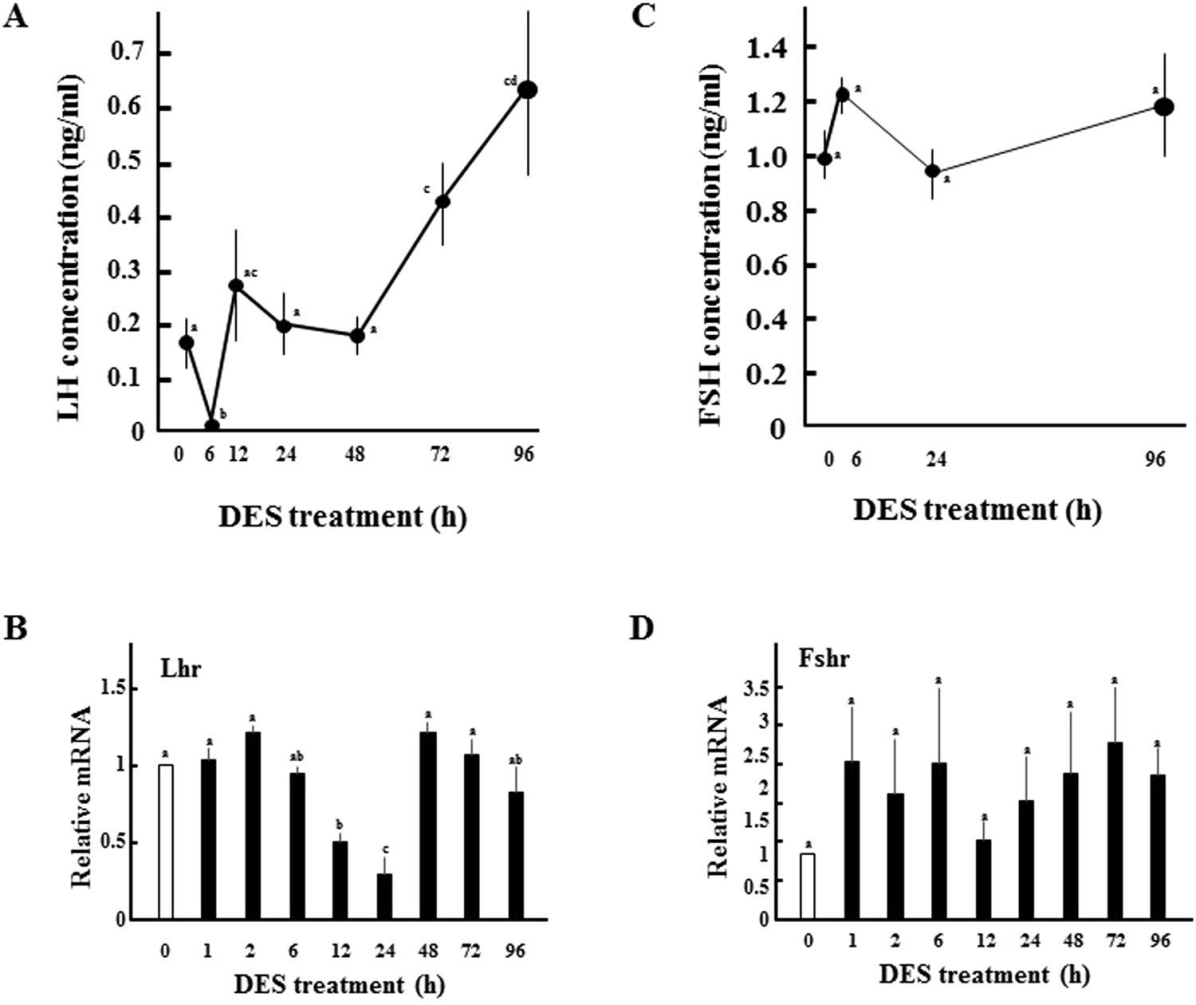
Effects of DES on serum gonadotropin levels and expression of ovarian cognate receptors. Concentrations of LH (**A**) and FSH (**C**) in immature female rats treated with DES for the indicated time. Data for each point are from five animals (the means ± SEM). Expression of ovarian LH receptor (LHR; **B**) and FSH receptor (FSHR; **D**) in immature rats treated with DES for the indicated time. Expression of each gene was analyzed by Q-PCR and normalized to 36B4 expression. Q-PCR data represent the mean ± SEM of at least four independent samples. Values marked by different letters are significantly different (*P* < 0.05).

**Figure 3:**
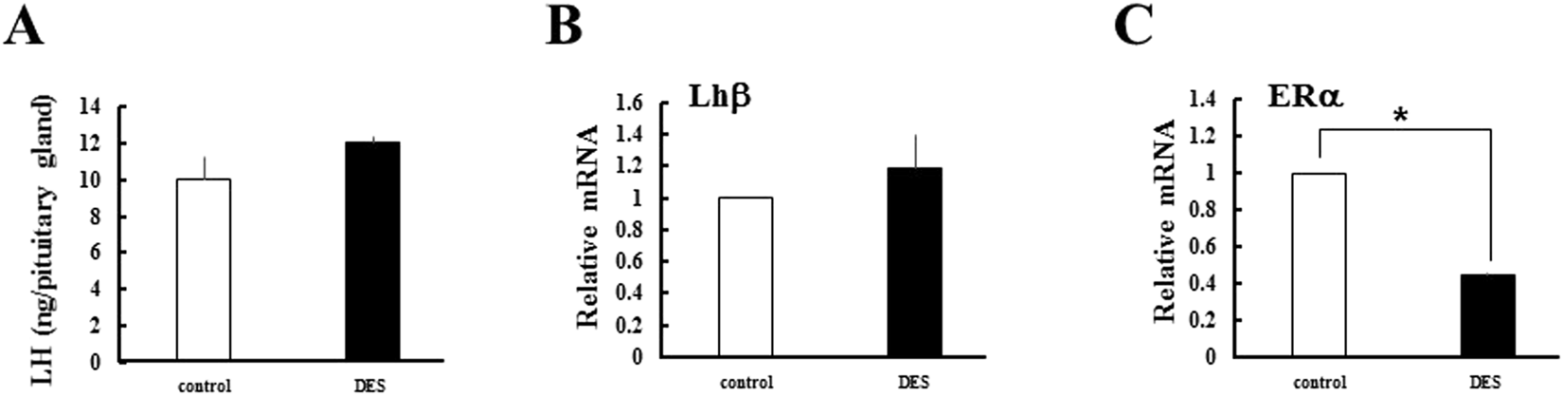
Effects of DES on pituitary LH content and gene expression. (**A**) Pituitary LH content in immature rats before and after 6 h treatment with DES. Data for each point are from three animals (the means ± SEM). Expression of pituitary Lhβ (**B**) and ERα (**C**) in immature rats before and after 6 h treatment with DES. Expression of each gene was analyzed by Q-PCR and normalized to 36B4 expression. Q-PCR data represent the mean ± SEM of at least three independent samples. Differences between groups are indicated by *P < 0.05.

As we previously reported^23^, serum LH levels were acutely decreased within 30 min after DES-treatment, and maintained minimal levels until 6 h later (Fig. 2A). Expression of LH content and Lhβ mRNA in pituitaries were unaffected by DES-treatment (Fig. 3A,B). LH levels recovered after 12 h, and increased further from 48 h to 96 h (Fig. 2A). Pituitary ERα expression was reduced by DES-treatment, and further decreased with the elevation of serum LH levels (Fig. 3C, Supplementary Fig. S3). On the other hand, LH levels were almost constant in control animals (Supplementary Fig. S2). Expression of ovarian Lhr was significantly decreased at 12 h and 24 h, although it returned to initial levels at 48 h (Fig. 2B). In contrast to LH, serum FSH concentrations remained almost constant (Fig. 2C). While expression of Fshr was increased at various time points, this was not statistically significant (Fig. 2D). Collectively, these results indicate that DES inhibits ovarian LH actions by repressing both ligand secretion from the pituitary and ovarian receptor expression, despite the high possibility for recovery at later times. FSH secretion and its ovarian actions appeared to be uncompromised by DES treatment.

### DES dramatically alters steroidogenesis-related gene expression and serum steroid hormone concentrations

**Figure 4:**
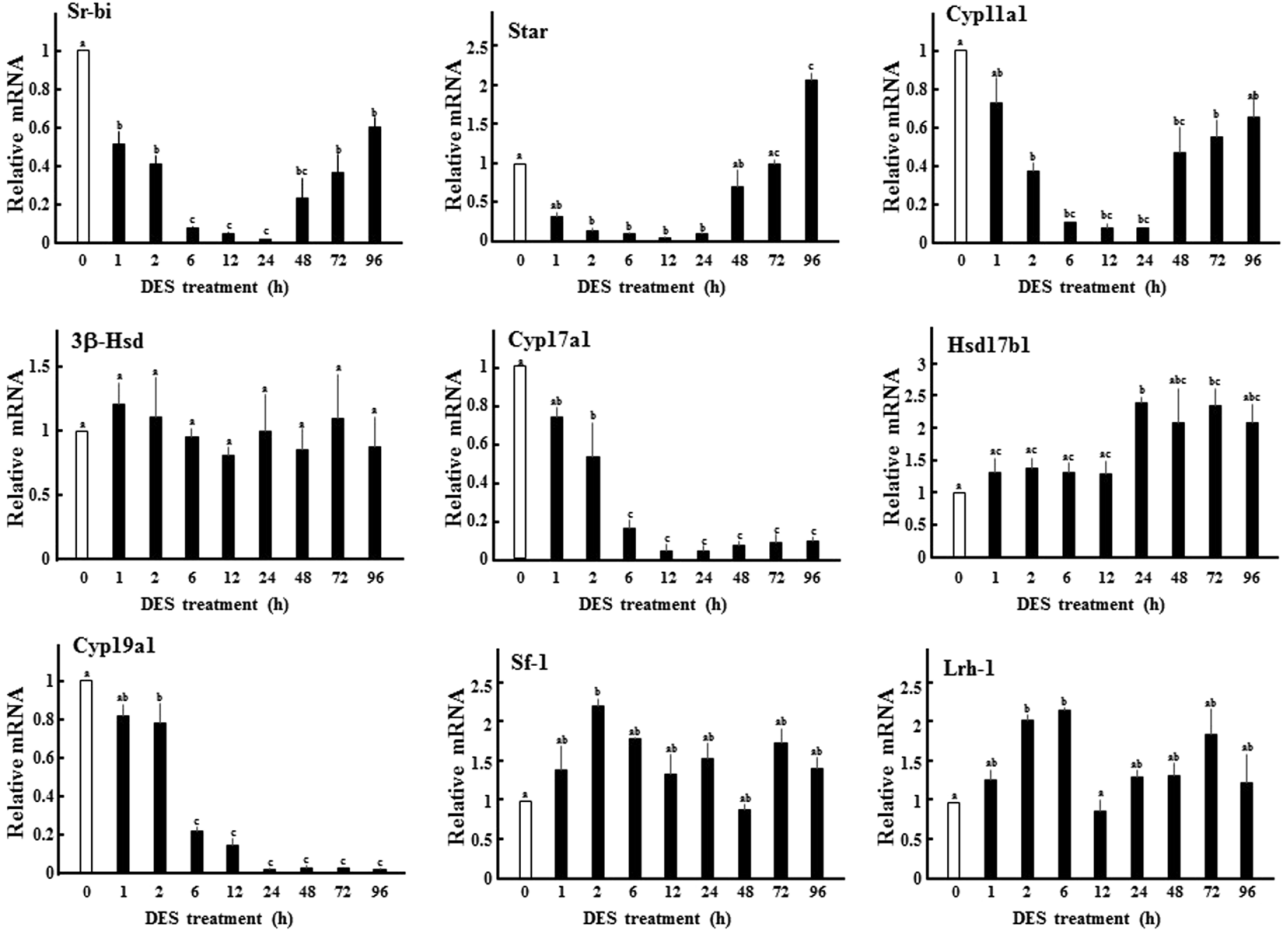
Effects of DES on expression of ovarian steroidogenesis-related genes. Expression of steroidogenesis-related genes in ovaries from immature rats treated with DES for the indicated time. Expression of each gene was analyzed by Q-PCR and normalized to 36B4 expression. Q-PCR data represent the mean ± SEM of at least four independent samples. Values marked by different letters are significantly different (*P* < 0.05).

**Figure 5:**
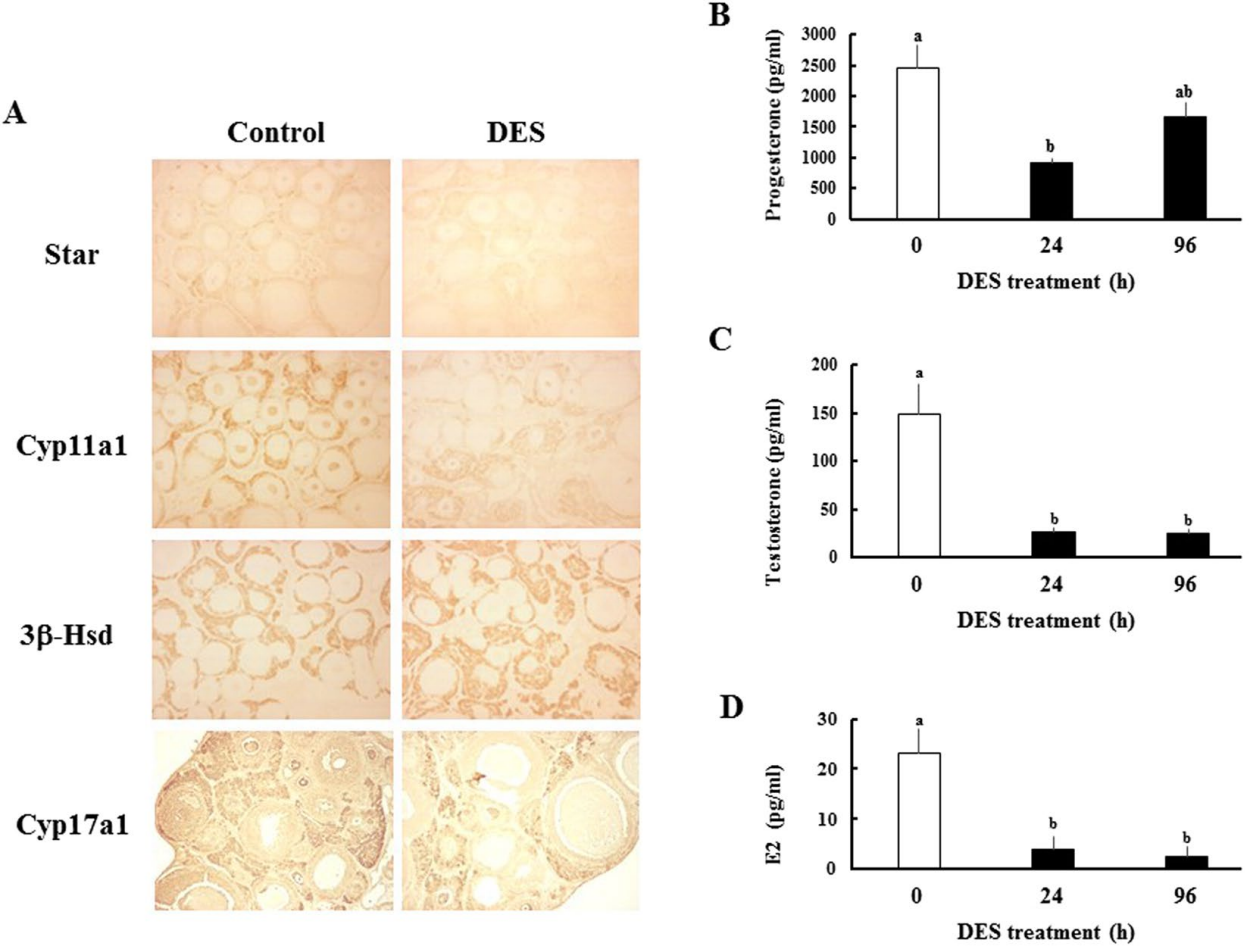
Effects of DES on the ovarian steroidogenesis-related proteins and the concentrations of serum steroid hormones. (**A**) Localization of Star, Cyp11a1, 3β-Hsd and Cyp17a1 proteins in immature ovaries before or 6 h after DES injection. Positive staining both for each protein was observed in theca cells. (**B–D**) Serum progesterone, testosterone and E2 levels in immature female rats treated with DES for indicated times. Data represent the mean ± SEM of at least 5 independent samples. Values marked by different letters are significantly different (*P* < 0.05).

With a reduction of serum LH levels, the expression of ovarian steroidogenic enzymes, such as Cyp11a1 and Cyp17a1, was reduced within 2 h and further decreased until 6 h (Fig. 4). At this time, steroidogenesis-related genes such as Scarb1 (Sr-bi) and Star were also decreased. Immunohistochemical analysis showed that these proteins are strongly expressed in theca cells, although their signals were reduced 6 h after DES administration (Fig. 5A). In contrast, expression levels of 3β-Hsd were unaffected by DES-treatment. In addition to altering steroidogenic genes in theca cells, expression of Cyp19a1 was decreased in a similar fashion in granulosa cells (Fig. 4). In contrast, Hsd17b1 expression remained at levels observed before DES-injection. These conditions were maintained until 24 h later. Following the recovery of serum LH concentrations and Lhr expression, most down-regulated genes began to increase from 48 h until their expression levels reached to nearly initial levels at 96 h. Star expression was significantly higher than that observed before DES-treatment. In addition, Hsd17b1 expression was significantly increased at some time points. However, Cyp17a1 and Cyp19a1 expressions remained repressed at these time points, indicating their expression is repressed by factors other than gonadotropin secretion. Notably, expression of Sf-1 and Lrh-1, transcriptional factors that regulate levels of steroidogenesis-related genes in theca and granulosa cells^6^, was not reduced by DES-treatment at any time. Expression of steroidogenesis-related genes was largely unaffected in sesame oil-injected animals during the experimental period (Supplementary Fig. 4).

Serum progesterone, testosterone and E2 concentrations were decreased to 37.5%, 11.5% and 16.8% of initial levels at 24 h, respectively (Fig. 5B–D). Consistent with the expression pattern of synthetic enzymes, progesterone levels were increased about two-fold at 96 h, whereas testosterone and E2 levels were maintained at lower levels.

### DES induces inducible nitric oxide synthase to repress Cyp19a1 expression in granulosa cells

**Figure 6:**
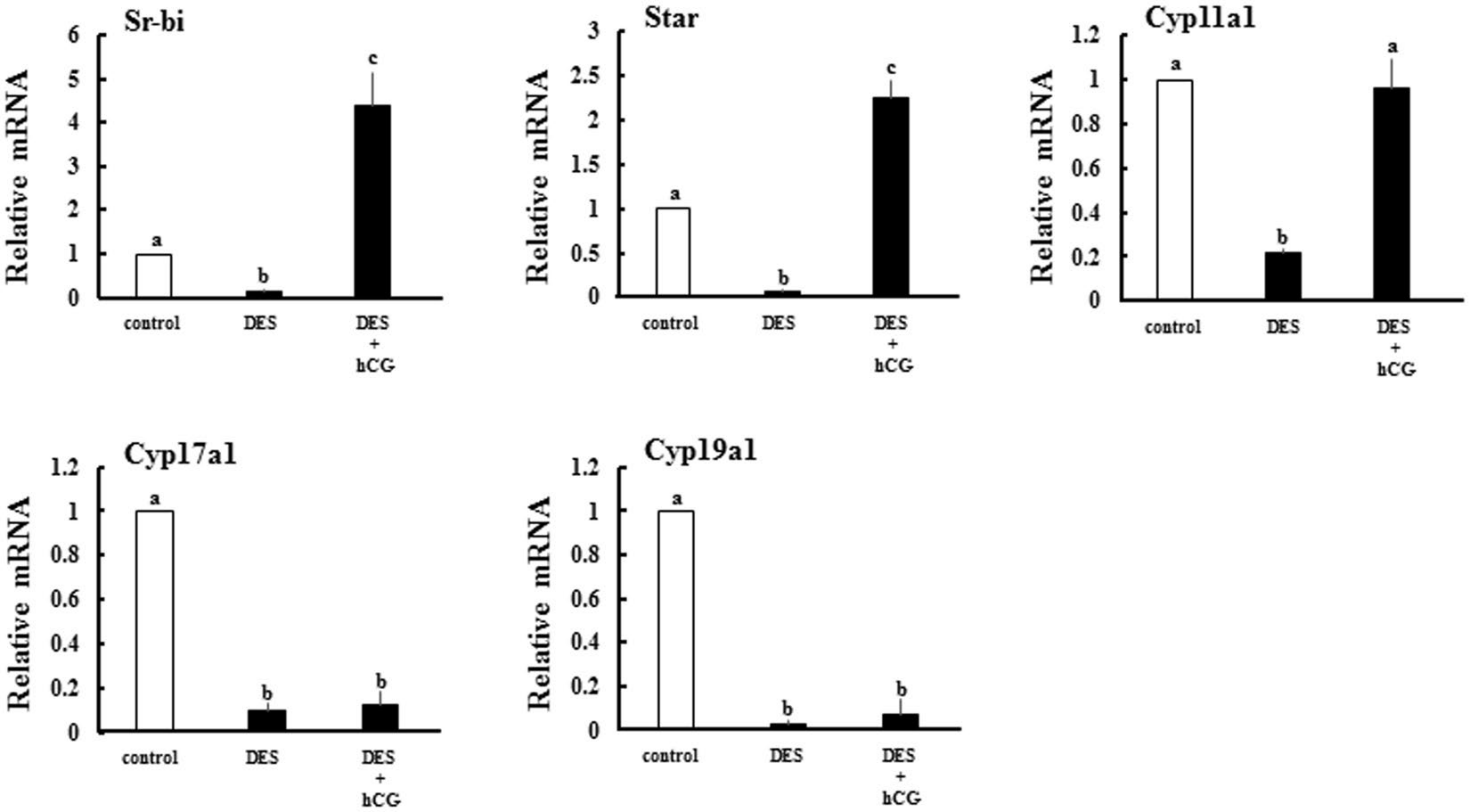
Effects of hCG on ovarian steroidogenesis-related gene expression in DES-treated animals. Steroidogenesis-related gene expression in ovaries from immature rats treated with vehicle, DES and hCG for 6 h. Expression of each gene was analyzed by Q-PCR and normalized to 36B4 expression. Q-PCR data represent the mean ± SEM of at least three independent samples. Values marked by different letters are significantly different (*P* < 0.05).

Suppression of Cyp17a1 and Cyp19a1 expression is likely caused by direct ovarian actions of DES, as this was recapitulated in hypophysectomized models (Supplementary Fig. S5). In addition, the expression of these genes was not recovered by co-treatment of human chorionic gonadotropin (hCG) and DES, although Sr-bi, Star and Cyp11a1 genes were up-regulated by hCG (Fig. 6). In fact, it was shown in knockout mice that ERα directly represses Cyp17a1 expression in theca cells^24^. However, the regulation of Cyp19a1 expression by estrogen remains to be revealed. It was reported in previous studies that nitric oxide (NO) is a strong repressor for Cyp19a1 activity in granulosa and luteal cells^25–28^. Thus, we investigated ovarian expression of NO synthase (NOS) in DES-treated animals.

**Figure 7:**
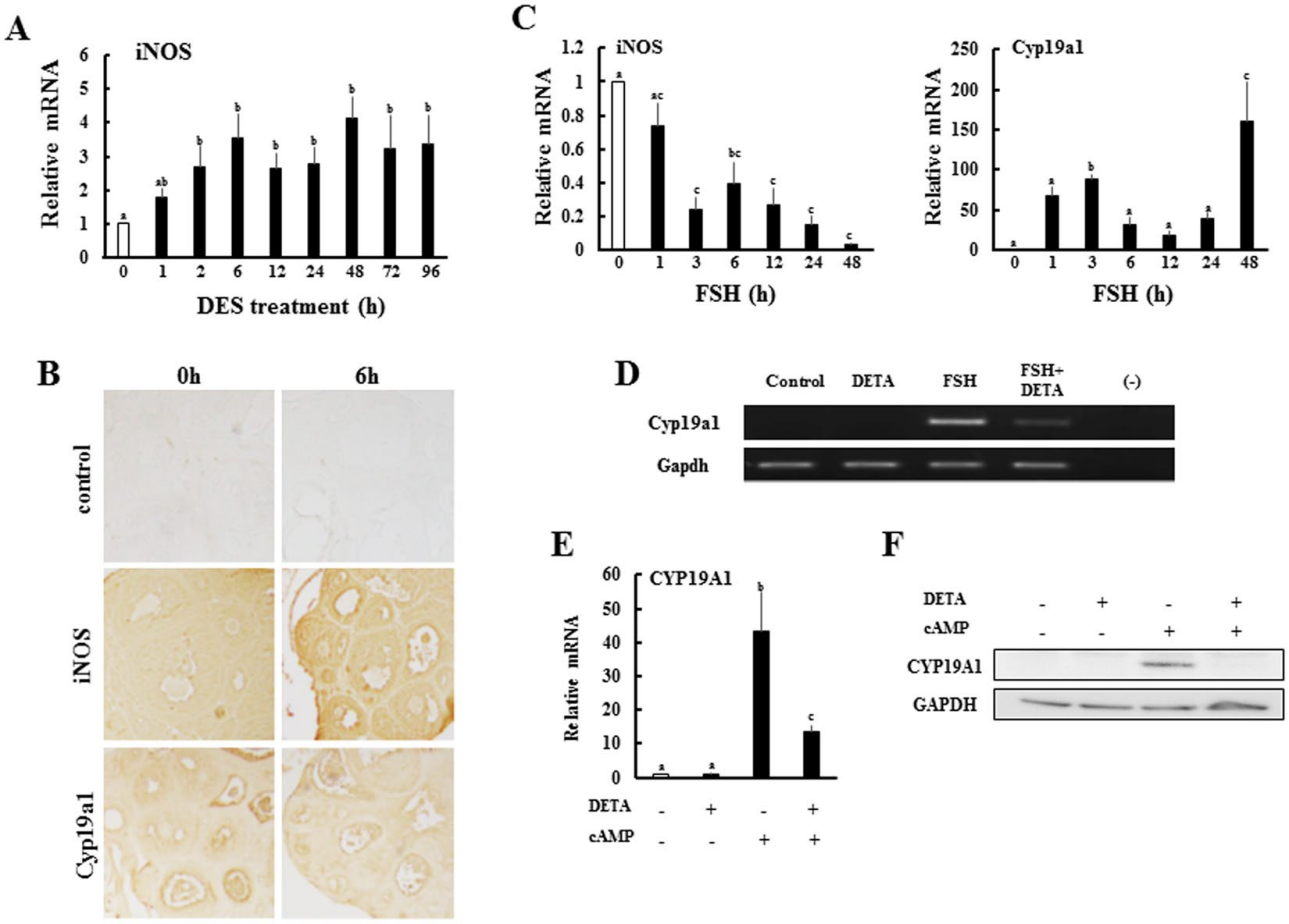
Effects of nitric oxide (NO) on Cyp19a1 expression in granulosa cells. (**A**) Expression of ovarian inducible nitric oxide synthase (iNOS) gene in DES-treated animals for the indicated times. Q-PCR data represent the mean ± SEM of at least four independent samples. Values marked by different letters are significantly different (P < 0.05). (**B**) Localization of iNOS and Cyp19a1 proteins in immature ovaries before (0 h) or after (6 h) DES injection. Both proteins were localized on granulosa cells. No staining was observed in control sections incubated with nonimmune serum. (**C**) Granulosa cells were isolated from immature DES-primed rats and treated with FSH (30 ng/ml) for the indicated times. Gene expression of each gene was measured by Q-PCR. Q-PCR data represent the mean ± SEM of at least four independent samples. Values marked by different letters are significantly different (*P* < 0.05). (**D**) Granulosa cells were isolated from immature DES-primed rats and treated with FSH (30 ng/ml) and DETA NONOate (50 μM) for 4 h. Gene expression of each gene was measured by RT-PCR. Uncropped images are shown in Supplementary Fig. S3. Effects of NO generator on expression of CYP19A1 mRNA (**E**) and proteins (**F**) in KGN cells. KGN cells were treated with 8-br-cAMP (1 mM) and DETA NONOate (50 μM) for 24 h. (**E**) CYP19A1 gene expression was measured by Q-PCR. Q-PCR data represent the mean ± SEM of at least four independent samples. Values marked by different letters are significantly different (*P* < 0.05). (**F**) Western blot analyses were performed with antibodies against CYP19A1 and GAPDH using lysates from KGN cells (30 μg protein) in each group. Uncropped blots are shown in Supplementary Fig. S3.

Among three types of NOS isoforms^29^, inducible NOS (iNOS) was induced by DES-treatment within 2 h and increased thereafter (Fig. 7A). Indeed, iNOS was expressed at significantly higher levels than initially observed levels, even after 96 h. Immunohistochemical analysis demonstrated that iNOS protein was mainly induced and localized in granulosa cells of secondary and antral follicles (Fig. 7B). In contrast, Cyp19a1 protein was markedly reduced by DES-treatment within granulosa cells in nearly all follicles.

Cyp19a1 is induced by FSH in granulosa cells isolated from DES-primed rats (Fig. 7C). In contrast, FSH-treatment robustly decreased iNOS expression. The NO generator DETA NONOate strongly repressed Cyp19a1 expression induced by FSH (Fig. 7D). It also inhibited induction of CYP19A1 expression by 8-br-cAMP in human granulosa cell tumor-derived KGN cells at both mRNA and protein levels (Fig. 7E,F). These results indicate that DES represses Cyp19a1 expression through NO production by induction of iNOS.

## Discussion

To investigate various phenomena occurring during granulosa cell differentiation, many researchers have used granulosa cells from immature rodents primed with E2 or DES. These estrogens strongly promote the proliferation of granulosa cells in immature follicles to provide large numbers of relatively undifferentiated granulosa cells for *in vitro* experiments^20–22^. These granulosa cells are known to be highly responsive to FSH and differentiate into the cells showing a preovulatory stage phenotype. However, potential effects of estrogens on the hypothalamic-pituitary-ovary axis have not been well evaluated in this model. In this study, we demonstrated that DES not only induces granulosa cell proliferation, but also affects various endocrine functions of ovary and pituitary tissues.

Ovarian E2 exerts both negative and positive feedback effects on LH secretion via ERα^30–32^. In most stages of the reproductive cycle, the estrogen/ERα pathway exerts negative feedback actions to reduce GnRH secretion from GnRH hypothalamic neurons, resulting in a reduction of LH production and secretion from the pituitary^30^. In addition, the estrogen/ERα pathway directly inhibits LH secretion from pituitary^31^. An initial acute reduction of serum LH concentrations in DES-treated rats represents this negative feedback of the estrogen/ERα pathway. As LH content and Lhβ expression were largely unaffected in the pituitaries of DES-treated animals, it is conceivable that DES suppressed LH secretion rather than LH production. However, this effect disappeared at 12 h, and LH levels progressively increased thereafter until 96 h. Attenuation of negative feedback by down-regulation of pituitary ERα expression might be one potentially important mechanism for this phenomenon. In addition to pituitary levels, there are also a few possibilities underlying this phenomenon at hypothalamus levels. One possibility is the positive effects of estrogen on LH secretion. In contrast to the early phase of follicular stages, increased E2 exerts positive feedback action on LH secretion (LH surge) by increasing GnRH release at later follicular stages^33^. As such, continuous DES administration potentially evoked such positive feedback effects. Another possibility is the pubertal rise of LH levels. A previous study reported that exogenous estrogen induces early puberty onset with elevated serum LH levels in prepubertal rats^34^. Kisspeptin 1 (Kiss1) is an important peptide hormone not only for increasing GnRH release both during LH surge and pubertal LH elevation, but also for reducing GnRH release in a negative feedback effect of the estrogen/ERα pathway^35–37^. As ERα differentially regulates Kiss1 expression within different parts of Kiss1 neurons between negative feedback/puberty and LH-surge^38–40^, it would be interesting to investigate the fluctuation of Kiss1 expression in DES-treated rats.

Steroidogenesis and the expression of steroidogenesis-related genes in theca cells are primarily under control of the LH/LHR pathway^2^. Consistent with this fact, serum concentrations of each steroid hormone and the expression of almost steroidogenesis-related genes decreased in response to a reduction of LH and LHR expression. Furthermore, the recovery of LH levels and LHR expression was accompanied by the restoration of steroidogenesis-related gene expression, with the exception of Cyp17a1. In addition, Cyp17a1 expression was not recovered by co-treatment with hCG and DES. These results suggest that Cyp17a1 is repressed by the direct ovarian actions of DES. In support of our results, Korach and colleague reported that intra-follicular ERα inhibits androgen synthesis in theca cells by repressing Cyp17a1 expression^24^. Ovarian follicles from ERα-null mice expressed markedly increased levels of Cyp17a1 and secreted significant higher amounts of androgens, compared with follicles from wild-type animals. These phenotypes of αERKO follicles are recapitulated in wild-type follicles by treatment with aromatase inhibitor or ER-antagonist. Taken together, this strongly suggests that Cyp17a1 and androgen production could be primary targets of EDCs in ovarian theca cells.

In addition to thecal steroidogenesis-related genes, Cyp19a1 expression in granulosa cells was repressed by DES, even though FSH secretion and FSHR expression were not compromised. Because DES-treatments did not inhibit the expression of other FSH-regulated genes, such as Hsd17b1 and inhibin-α (unpublished data), repression of Cyp19a1 expression was likely caused by the direct ovarian actions of DES. Previous studies suggest that NO is important for various phenomena during ovulation. Inhibition of NO generation by NOS inhibitors suppresses follicle rupture and oocyte maturation by LH-surge, resulting in a reduction of ovulation rate and normal development^41–43^. NOS inhibitors also enhance estrogen production by granulosa cells, as NO is involved in down-regulation of CYP19A1 activity and expression during ovulation^26, 27^. Among the three isoforms of NOS, endothelial NOS (eNOS) is induced and plays important roles at this phase. eNOS KO female mice exhibit overproduction of E2 and the reduced ovulation^44–46^. However, iNOS was the primary isoform induced in the ovary of DES-treated animals. Consistent with earlier reports^47^, iNOS was expressed in the granulosa cells of secondary follicles in immature ovaries. In addition to this early stage, iNOS was also induced in some granulosa cells of antral follicles by DES-treatment. In addition, CYP19A1 expression is up-regulated during the antral follicle stage by FSH signaling^3, 48, 49^. Then, it is reasonable that DES repressed this induction of CYP19A1 by NO generation through iNOS. This phenomena likely contributes to maintain the undifferentiated conditions of granulosa cells in DES-primed rodent ovary. In contrast to DES administration, FSH signaling acutely suppressed iNOS expression. These results suggest the possibility that iNOS is not only involved in the repression of CYP19A1 expression by DES, but also in the suppression of precocious CYP19A1 expression in granulosa cells during the early follicle stage under physiological conditions. This can be supported by the facts that E2 levels are increased during proestrous stage in iNOS KO mice^44^.

It is well-known that NO can modulate the activity of various enzymes by binding to iron and other enzyme components^29, 50^. Indeed, CYP19A1 protein is inhibited by the direct binding of NO to heme and other portions^25^. In addition to the direct binding, transcriptional regulation should also be essential for the inhibition of CYP19A1 activity by NO. This may be even more important in granulosa cells, because NO inhibited induction of the *Cyp19a1* gene by FSH from an early time point (4 h). In support of this hypothesis, Ishimaru *et al*. also reported that NO generator completely inhibited FSH-induced Cyp19a1 activity by cyclic guanosine monophosphate (cGMP) in rat granulosa cells^28^. Although NO stimulates intracellular cGMP production through activation of soluble guanylyl cyclase, this signaling has often been reported as irrelevant to the inhibition of CYP19A1 by NO^26, 51, 52^. Therefore, in addition to inhibition of enzyme activities by direct binding, it is plausible that other mechanisms are important for the repression of CYP19A1 in granulosa cells. However, this group also demonstrated that cGMP reduced the elevation of intracellular cAMP concentrations by FSH and forskolin in granulosa cells, resulting in the inhibition of Cyp19a1 activity^28^. This is inconsistent with our result, because NO strongly inhibited the cAMP-induced Cyp19a1 expression. Such a discrepancy might be derived from investigating the different outputs of Cyp19a1 in these studies, namely gene expression and enzyme activity. Moreover, incubation times with NO donor (72 h) previous used was much longer than employed in our study. Therefore, NO likely exerts various inhibitory effects on Cyp19a1 to repress the estrogen production in granulosa cells. In future studies, it is necessary to reveal mechanisms for the transcriptional regulation of *Cyp19a1* gene by NO.

**Figure 8:**
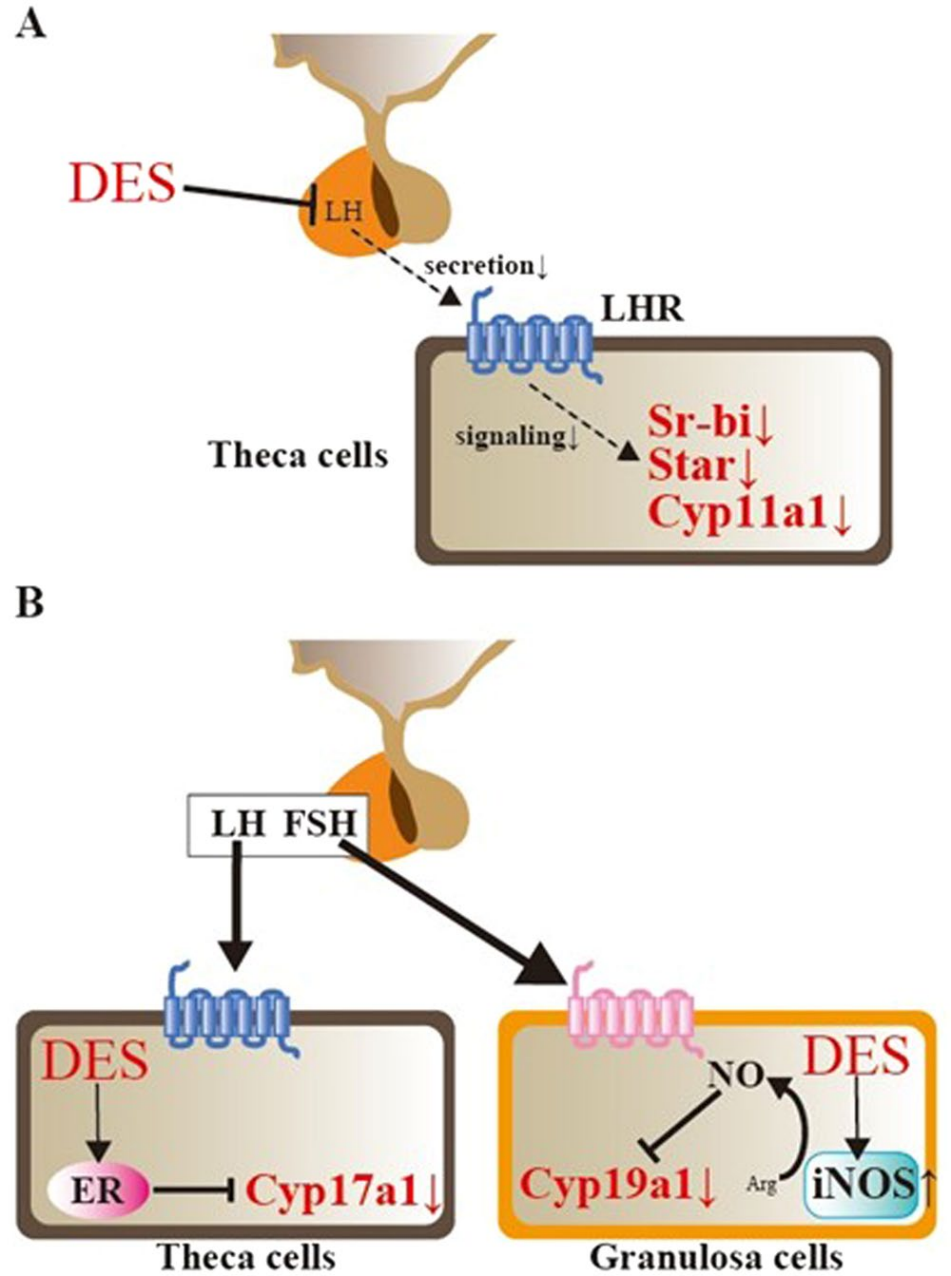
Proposed model of the effects of DES on ovarian steroidogenesis. (**A**) During the initial phase, DES inhibits both LH secretion from the pituitary and expression of steroidogenesis-related genes in theca cells. (**B**) After recovery of LH/LHR signaling, theca cell androgen and granulosa cell estrogen production continued to be suppressed by reduced Cyp17a1 and Cyp19a1 expression via direct actions of DES on ovary.

In summary, we demonstrated that DES initially suppresses steroidogenesis in theca cells by affecting LH secretion and LH/LHR signaling in immature rats (Fig. 8A). In addition, it also inhibits estrogen production by repressing Cyp19a1 expression via the induction of *iNOS* gene. Progesterone production was recovered by an abatement of the effects of DES on the LH/LHR system at later time points. However, androgen and estrogen production continued to be suppressed by reduced Cyp17a1 and Cyp19a1 expression (Fig. 8B). Our results strongly suggest that in addition to LH secretion in the pituitary, sex steroid production by these genes are primary targets of EDCs in the prepubertal ovary and, thus, could be important points for estimating the effects of EDCs on female reproduction.

## Methods

### Animals and hormone assay

Normal or hypohysectomized immature Wistar female rats (21–28 d old) were purchased from Japan SLC, Inc. (Shizuoka, Japan). At all times, animals were treated according to NIH guidelines. All animal experiments were approved by the institutional animal care and use committee of the University of Fukui and the Asahikawa Medical University. Animals were treated subcutaneously with 2 mg DES in 0.2 ml sesame oil once daily for 4 consecutive days. 15 IU hCG (Sankyo, Tokyo, Japan) was intraperitoneally injected at the same time as DES-treatment. After collection of serum and pituitary samples, serum concentrations of LH (Endocrine Technologies, Inc., Newark, CA, USA), FSH (Shibayagi Co, Ltd., Gunma, Japan), progesterone (Cayman Chemical Company, Ann Arbor, MI, USA), testosterone (Cayman Chemical Company), estradiol (Cayman Chemical Company) and pituitary LH concentrations (Shibayagi Co, Ltd) were determined by ELISA and EIA as described^23, 53^.

### Cell culture, transfection and luciferase assay

Granulosa cells were obtained from immature Wistar female rats that received an injection of 2 mg DES in 0.2 ml sesame oil once daily over four consecutive days. Granulosa cell culture was performed as described previously^54, 55^. At all times, the animals were treated according to NIH guidelines. Granulosa cells were cultured in DMEM/Ham’s F-12 supplemented with 0.1% BSA on collagen-coated plates.

COS-7 cells were cultured in Dulbecco’s modified Eagle’s medium (DMEM) with 10% fetal calf serum (FCS). KGN cells (kindly provided by Dr. Toshihiko Yanase, Fukuoka University, Fukuoka, Japan) were cultured in DMEM/Ham’s F-12 medium with 10% FCS. COS-7 cells were transfected using Lipofectamine LTX (Life Technology, CA, USA). After 24 h of transfection, the cells were treated with vehicle, E2 or DES for 24 h. Luciferase assays were performed as described previously^53–56^. Each data point represents the mean of at least four independent experiments.

### Plasmids

pSG424-ERα (amino acids 256–600) and pSG424-ERβ (amino acids 181–530) were constructed by inserting the corresponding cDNAs of rat ERα and ERβ to pSG424 vector. Other plasmids were described previously^54^.

### RT-PCR and Quantitative (Q)-PCR

Total RNA from ovaries, pituitaries, granulosa cells and KGN cells was extracted using the Tripure reagent (Roche, Basel, Switzerland). RT-PCR and Q-PCR was performed as described^57^. The RT-PCR products were electrophoresed on a 1.5% (w/v) agarose gel, and the resulting bands were visualized by staining with ethidium bromide. The primers used have been described previously^54^ and are outlined in Supplementary Table S1.

### *In situ* hybridization

*In situ* hybridization was performed as described^58^. Rat ovaries were fixed in freshly prepared 4% paraformaldehyde, dehydrated with ethanol, cleared in xylene, and embedded in paraffin. Sections (5 μm thick) were mounted on (3-aminopropyl) triethoxysilane-coated glass slides for *in situ* hybridization. The sections were deparaffinized, rehydrated, and acetylated before hybridization. Rat ERα and ERβ cDNA fragments were subcloned into the pBluescript (Agilent Technologies, Inc. CA, USA). Antisense or sense ^35^S-CTP-labeled RNA probes were synthesized using T7 or T3 RNA polymerase. Hybridization with the ^35^S-labeled cRNA probes was performed for 6 h, and the sections were then washed under conditions of high stringency and autoradiographed using an NTB2 emulsion (Eastman Kodak Co., Rochester, NY, USA).

### Immunohistochemistry

Immunohistochemistry was performed as described previously^54, 56^. Briefly, rat ovaries were fixed in 4% paraformaldehyde solution, dehydrated in a graded ethanol series, and embedded in paraffin wax. Sections of 7 µm thickness were subjected to an antigen retrieval technique with HistoVT One (Nacalai Tesque Inc., Kyoto, Japan) or Dako Target Retrieval Solution, pH 9 (Dako Denmark A/S, Glostrup, Denmark), and treated with anti-StAR (kindly given by Dr. W. Miller, University of California, San Francisco^59^), anti-P450scc (C-16, Santa Cruz, CA, USA), anti-3β-HSD (kindly provided by Dr. A. Payne, Stanford University Medical Center), anti-P450c17 (kindly provided by Dr. D. Hales), CYP19A1 (MCA20775; AbD Serotec, Oxford, UK) and anti-iNOS (K13-A, Novus Biologicals, Littleton, CO, USA) antibodies. Then, they were developed using a Vectastain Elite ABC kit (Vector Laboratories, Burlingame, CA, USA).

### Western blotting analysis

Extraction of total proteins from cultured cells and subsequent quantification were conducted as described previously^57^. Equal amounts of protein (30 μg) were resolved using 10% SDS-PAGE and transferred to polyvinylidene difluoride membranes. Western blot analyses of CYP19A1 and GAPDH were performed with antibodies directed against CYP19A1 (MCA20775; AbD Serotec, Oxford, UK) and GAPDH (14C10; Cell Signaling Technology, Inc., Massachusetts, USA). Enhanced chemiluminescence western blot reagents (Bio-Rad Laboratories Inc., Hercules, CA, USA) were used for detection.

### Statistical analysis

Data are presented as the mean ± SEM. Differences between groups (*P* < 0.05) were assessed by the Student’s *t*-test or one-way ANOVA followed by Tukey’s multiple comparison tests using EZR (Saitama Medical Center, Jichi Medical University, Saitama, Japan)^60^, which is a graphical user interface for R (The R Foundation for Statistical Computing, Vienna, Austria).

## Electronic supplementary material

Supplementary information
