# Supplementary material for: Diethylstilbestrol administration inhibits theca cell androgen and granulosa cell estrogen production in immature rat ovary
Source: Sci Rep. 2017 Aug 21;7:8374. doi: 10.1038/s41598-017-08780-7 (PMC5567288; doi:10.1038/s41598-017-08780-7)
Supplement: Supplementary file 1 — Supplementary information [file 41598_2017_8780_MOESM1_ESM.pdf]

Diethylstilbestrol administration inhibits theca cell androgen and granulosa cell estrogen production in immature rat ovary

Yoshitaka Imamichi, Toshio Sekiguchi, Takeshi Kitano, Takashi Kajitani, Reiko Okada, Yoshihiko Inaoka, Kaoru Miyamoto, Junsuke Uwada, Satoru Takahashi, Takahiro Nemoto, Asuka Mano, Md Rafiqul Islam Khan, Md Tariqul Islam, Koh-ichi Yuhki, Hitoshi Kashiwagi, Fumitaka Ushikubi, Nobuo Suzuki, Takanobu Taniguchi and Takashi Yazawa

Supplementary materials:

Supplementary Table S1. Primers used in each experiment.

Supplementary Fig. S1. Effects of DES on the expression of estrogen receptors in immature rat ovaries.

Supplementary Fig. S2. Ovarian histology and serum LH concentrations in immature control rats.

Supplementary Fig. S3. Effects of DES on the expression of ERα in the pituitaries of immature rats.

Supplementary Fig. S4. The expression of each gene in the ovaries of immature control rats.

Supplementary Fig. S5. Effects of DES on the expression of each gene in the ovaries of hypophysectomized immature rats.

Supplementary Table S1. Primers used in each experiment.

PCR primers	Forward primers	Reverse primers
**RT-PCR**		
rat Cyp17a1	F-tgaattcagacaacaacaacagc	R-tctgcgtgggtgtaatgagatgg
rat Cyp19a1	F-cctggacgaaagttctattg	R-gaagcaacatgacgtacaga
rat iNOS	F-tccaacctgcaggtcttcgatgc	R-ggaccagccaaatccagtctgc
rat ERα	F-gctaccattatggggtctggt	R-atctgctaggttggtcaataa
**Q-PCR**		
human CYP19A1	F-atgaaagctctgtcaggccc	R-tcaacacgtccacatagccc
rat Lhr	F-attgacactctgcttaactttccatct	R-tggccatgaggtactcatgatct
rat Fshr	F-catcactgtgtccaaggqcca	R-tgcggaagttcttggtgaaaa
rat Cyp17a1	F-actgagggtatcgtggatgc	R-tcgaacttctccctgcactt
rat Hsd17b1	F-gttatgagcaagccctgagc	R-gcggtttgtggagaagtagc
rat Sf-1	F-ttctcctgacatctccttcctgat	R-agggcaagcaacagctcttc
rat Lrh-1	F-tcctggttaccggagaacac	R-tgaagggaacggagtctcac
rat iNOS	F-gaccagaaactgtctcacctg	R-cgaacatcgaacgtctcaca
rat ERα	F-ccgccttctacaggtccaat	R-gaaagccttgcagccttcac
rat ERβ	F-gaagctgaaccacccaatgt	R-caatcatgtgcaccagttc
rat Lhβ	F-tgagcccaagtgtggtgtg	R-ggcagctggcagtactcgaa

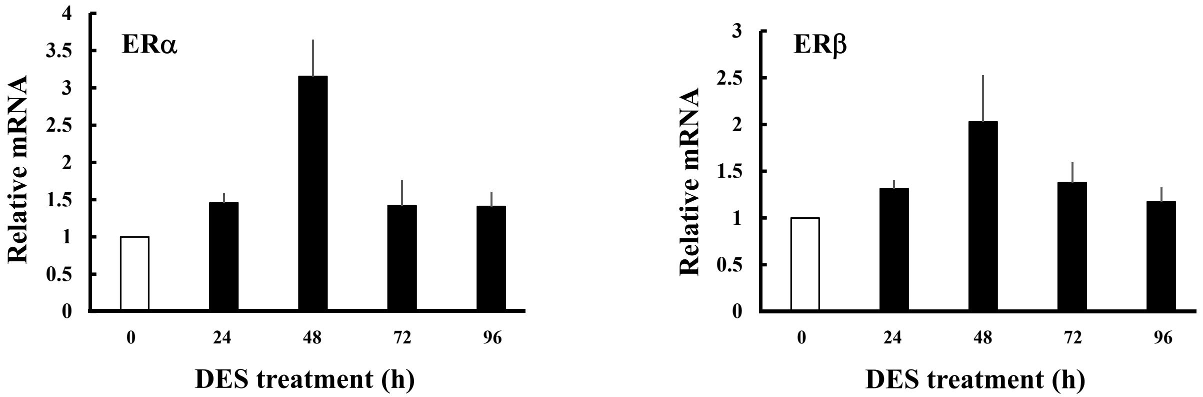

Supplementary Fig. S1 Effects of DES on the expression of estrogen receptors in immature rat ovaries. Animals were treated with DES, and ovaries were removed at indicated time. mRNA expression of each gene was analyzed by Q-PCR.

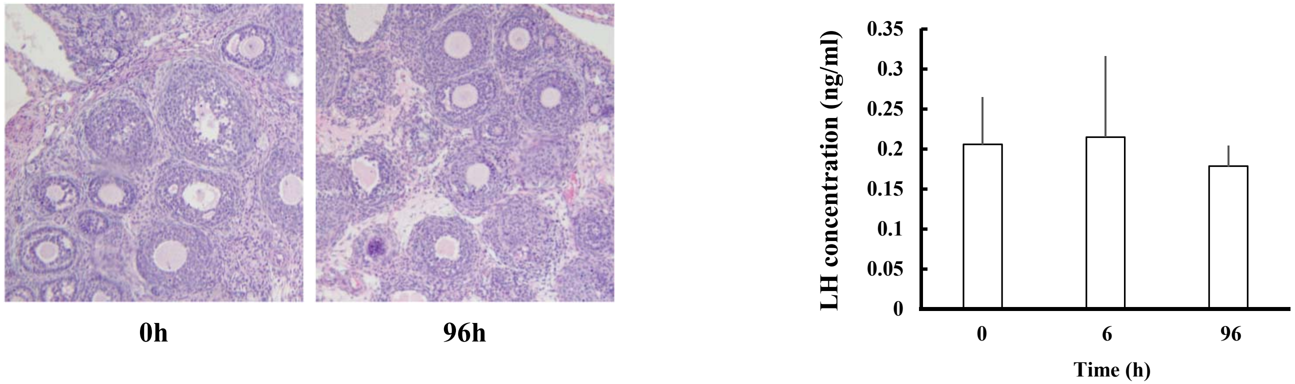

Supplementary Fig. S2 Ovarian histology and serum LH concentrations in immature control rats. Animals were injected with sesame oil, and ovaries and serum were collected at indicated time.

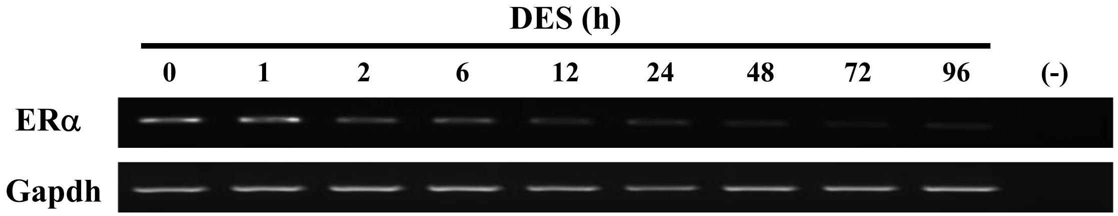

Supplementary Fig. S3 Effects of DES on the expression of ERα in the pituitaries of immature rats. Animals were treated with DES, and pituitaries were removed at indicated time. mRNA expression of each gene was analyzed by RT-PCR.

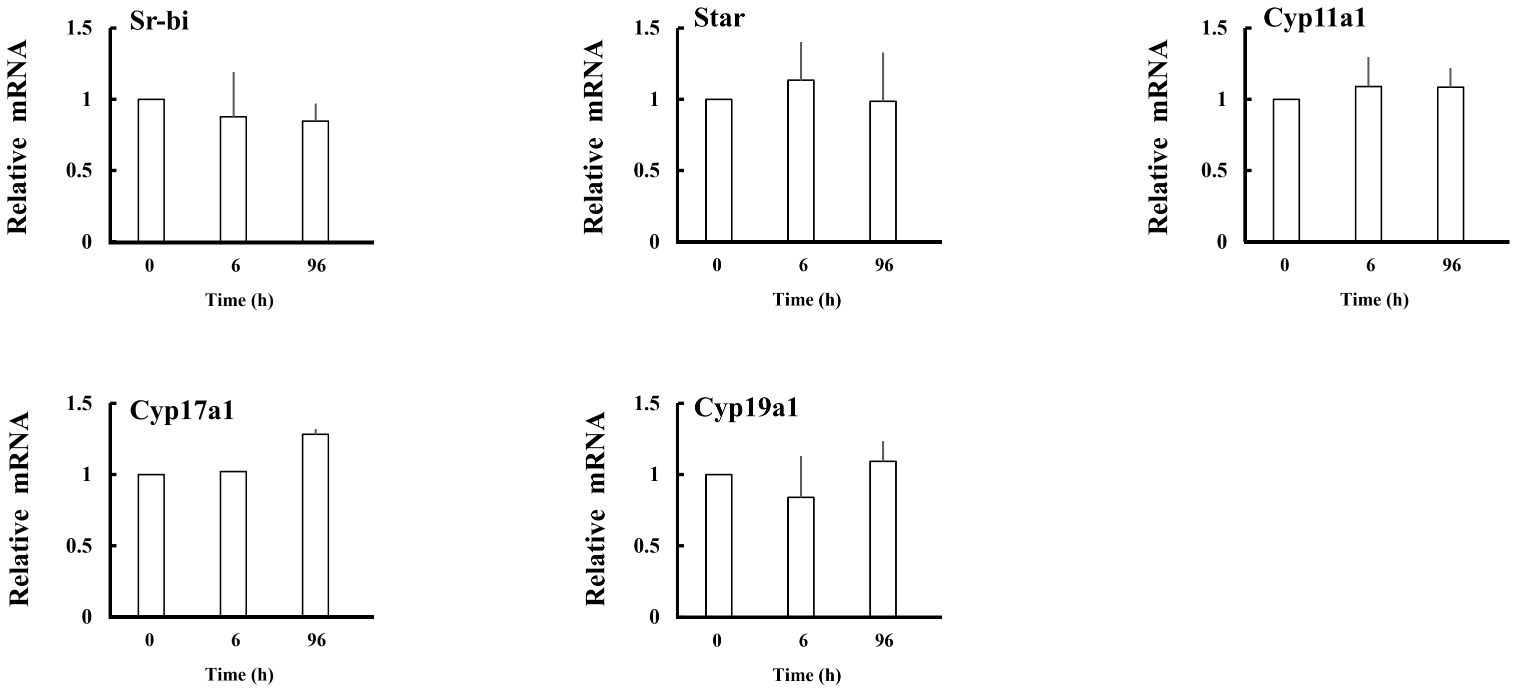

Supplementary Fig. S4 The expression of each gene in the ovaries of immature control rats. Animals were injected with sesame oil, and ovaries were removed at indicated time. mRNA expression of each gene was analyzed by Q-PCR.

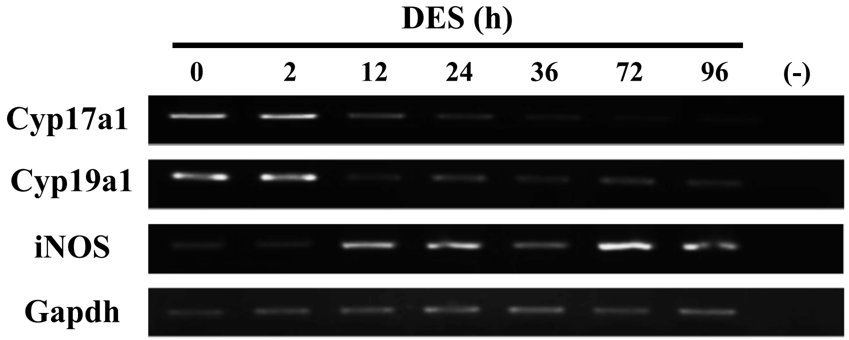

Supplementary Fig. S5 Effects of DES on the expression of each gene in the ovaries of hypophysectomized immature rats. Animals were treated with DES, and ovaries were removed at indicated time. mRNA expression of each gene was analyzed by RT-PCR.
